# Dynamic DNA Methylation Changes of *Tbx21* and *Rorc* during Experimental Autoimmune Uveitis in Mice

**DOI:** 10.1155/2018/9129163

**Published:** 2018-09-04

**Authors:** Yiguo Qiu, Yunyun Zhu, Hongsong Yu, Chunjiang Zhou, Aize Kijlstra, Peizeng Yang

**Affiliations:** ^1^The First Affiliated Hospital of Chongqing Medical University, Chongqing Key Laboratory of Ophthalmology, Chongqing Eye Institute, Chongqing, China; ^2^University Eye Clinic Maastricht, Maastricht, Netherlands

## Abstract

The key transcription factors of T helper cell subpopulations, including T-bet, GATA3, ROR*γ*t, and Foxp3 are involved in various autoimmune diseases. Whether methylation of these master transcription factors is associated with the development of experimental autoimmune uveitis (EAU) and the possible epigenetic regulatory mechanisms involved has however not yet been addressed. In our study, significant methylation changes in both *Tbx21* and *Rorc* were observed in one CpG site in the retinas of EAU mice. Two CpG sites of *Tbx21* and one CpG site of *Rorc* showed significant dynamic methylation changes in the RPE-choroid complex during EAU. The mRNA expressions of *Tbx21* and *Rorc* in both the retinas and RPE-choroid complexes correlated with the methylation changes at the various time points during EAU development. The methylation changes were associated with the production of the Th1/Th17 cells' signature cytokines, IFN-*γ* and IL-17. Dynamic changes in mRNA expression of DNA methyltransferases (DNMT1) were also noted, which may be related to the observed methylation changes of these genes. The present study provides evidence that DNA methylation of *Tbx21* and *Rorc* may be associated with the development of EAU. DNMT1 activation may have an important effect on regulating DNA methylation dynamics.

## 1. Introduction

Uveitis is an inflammatory and sight-threatening ocular disease which occurs worldwide and predominantly affects the working age population [1, 2]. It is defined as an intraocular inflammation of the uveal tract, although other intraocular structures such as the retina and vitreous may also be involved. Experimental autoimmune uveitis (EAU) is a classical uveitis animal model, which has been widely used to study human uveitis [3]. A specific retinal antigen, such as interphotoreceptor retinoid-binding protein (IRBP), can be used to induce EAU in susceptible mouse strains [4]. EAU induced by the IRBP_161–180_ peptide in a highly susceptible B10R.III mouse strain shows severe inflammation involving both the anterior and posterior segments of the eye and closely resembles the clinical abnormalities seen in human panuveitis [5].

Accumulating evidence has shown the importance of Th1, Th2, Th17, and Treg lymphocyte subsets in the pathogenesis of clinical uveitis [6–9]. T-bet (also known as Tbx21), GATA3 (GATA binding protein 3), ROR*γ*t (retinoid-related orphan receptor *γ*t, also known as Rorc), and Foxp3 (transcription factor forkhead box protein 3) are specific key transcription factors for these Th cell subsets [10, 11]. Previous studies showed that high gene copy number variations (CNVs) of Rorc conferred risk for ocular Behcet's disease (BD) and low CNVs of *Foxp3* predisposed to BD in female patients [12]. Moreover, high gene copy numbers of *Tbx21* and *Gata3* were found to correlate with higher susceptibility to acute anterior uveitis (AAU) and a high copy number of Foxp3 was associated with disease susceptibility in female AAU [13]. In addition, aberrant expressions of *Tbx21*, *Gata3*, and *Foxp3* have been observed in experimental autoimmune uveitis models [14–16].

DNA methylation is the most studied epigenetic modification. It is regulated by specific catalytically active enzymes, DNA methyltransferases (DNMTs), including DNMT1, DNMT3a, and DNMT3b, which have different roles either in maintaining methylation during DNA replication (DNMT1) or as de novo DNA methylators (DNMT 3a/3b) [17, 18]. DNA methylation is a well-established epigenetic means of gene regulation in the immune system [19]. Altered DNA methylation levels have been shown to be associated with various autoimmune diseases, including systemic lupus erythematosus (SLE) [20, 21], rheumatoid arthritis (RA) [22], multiple sclerosis (MS) [23], type 1 diabetes mellitus (T1DM) [24, 25], ocular Behcet's disease [26], and Vogt-Koyanagi-Harada (VKH) disease [27]. Recent observations suggest that specific methylation changes of the Th cell subset transcription factors may be involved in autoimmune disease [28–30]. An aberrant change of the *Foxp3* methylation level has been observed in RA patients [28], as well as in fulminant type 1 diabetes (FT1D) [29] patients, and a recent study from our group showed a higher methylation level of *Gata3* in patients with VKH uveitis [30].

In addition, dynamic changes in DNA methylation have been noticed in association with development and other pathophysiological processes [31, 32]. However, to the best of our knowledge, the DNA methylation dynamics of *Tbx21*, *Gata3*, *Rorc*, and *Foxp3* during the development of autoimmune disease has not yet been studied and was therefore the subject of our study, whereby we chose uveitis as a disease model. The results show significant methylation changes in *Tbx21* and *Rorc* in the inflamed ocular tissues of mice undergoing EAU, and we provide evidence that DNMT1 may play an important role in regulating DNA methylation of these two transcription factors.

## 2. Materials and Methods

### 2.1. Ethics Statement and Experimental Animals

All the experiments were conducted on the basis of the ARVO Statement for the Use of Animals in Ophthalmic and Vision Research. The Ethics Committee of the First Affiliated Hospital of Chongqing Medical University approved all the protocols of this study. All efforts were made to minimize animal discomfort and stress. B10.RIII mice were purchased from Jackson Laboratory (Bar Harbor, USA) and mice at the age of 6 to 8 weeks were used in the present study. Animals were housed at the Animal Care Service of Chongqing Medical University under specific pathogen-free conditions with a 12-12-hour light-dark cycle.

### 2.2. Induction of EAU

Interphotoreceptor retinoid binding protein (IRBP) peptide _161–180_ (IRBP_161–180_, SGIPYIISYLHPGNTILHVD) was synthesized by Shanghai Sangon (Shanghai, China). EAU was induced as described previously [33]. In brief, mice were immunized subcutaneously at the base of the tail and both thighs with 50 *μ*g human IRBP _161–180_ peptide in 100 *μ*l PBS, emulsified 1 : 1 (vol/vol) in complete Freund's adjuvant (CFA, Sigma-Aldrich, St. Louis, USA) supplemented with 1.0 mg/ml mycobacterium tuberculosis strain. A total of 200 *μ*l emulsion was injected per mouse.

### 2.3. Collection of the Retinas and the RPE-Choroid Complexes

Inflammation of the anterior segment was checked every two days from the 7th day to the 14th day (the peak of the inflammation) to confirm that the mice successfully developed EAU. During this time period, the eyes of the mice showed a gradual increase of inflammatory signs, including corneal edema, conjunctival hyperemia, hypopyon, and posterior synechiae as we described previously [33]. The retinas and RPE-choroid complexes were collected at day 0 (naïve), the 7th day, the 14th day, the 21st day, and the 28th day after IRBP immunization.

### 2.4. DNA Preparation and Bisulfite Treatment

QIAamp DNA Mini Kit (Qiagen, Valencia, USA) was used for the DNA extraction of the retinas and RPE-choroid complexes as the manufacturer's instruction. EZ DNA Methylation Kit (Zymo Research, Irvine, USA) was employed to perform the bisulfite conversion of genomic DNA (800 ng) with the following cycling conditions repeated for 20 cycles: 95°C for 30 seconds followed by 50°C for 15 minutes. Then the bisulfate DNA was used for further assay.

### 2.5. Quantitative Methylation Analysis of T-bet, GATA3, ROR*γ*t, and Foxp3 by the Sequenom MassARRAY EpiTYPER System

The MassARRAY EpiTYPER (Sequenom Inc., San Diego, USA) was used to detect the DNA methylation levels of *Tbx21*, *Gata3*, *Rorc*, and *Foxp3* using the following procedure. The PCR amplification was initiated with an incubation at 95°C for 4 minutes, and then denaturation was performed for 45 cycles at 95°C for 20 seconds, followed by annealing at 60°C for 30 seconds and extension at 72°C for 1 minute, and then finally incubated at 72°C for 3 minutes. The primers designed for methylation analysis are shown in Table 1. The CpG sites of *Tbx21* between 1806 and 1998, the CpG sites of *Gata3* between 895 and 1217, the CpG sites of *Rorc* between 1459 and 1739, and the CpG sites of *Foxp3* between 1496 and 1848 from the transcription start site (TSS) were detected according to the designed primers. The location information of methylation detection primers and CpG islands for each gene are shown in Table S1 and Figure S1. Subsequently, shrimp alkaline phosphatase (SAP) (Sequenom Inc.) was employed to dephosphorylate the unincorporated dNTPs at 37°C for 20 minutes, followed by 85°C for 5 minutes. Then the MassCLEAVE reaction was performed according to the manufacturer's instructions at 37°C for 3 hours. A MassARRAY™ Nanodispenser (Sequenom Inc., San Diego, USA) was used to dispense the end-product into a SpectroCHIP (Sequenom Inc., San Diego, USA). The spectra were acquired with a MassARRAY compact matrix-assisted laser desorption ionization time-of-flight (MALDI-TOF) mass spectrometer (Sequenom Inc., San Diego, USA). The EpiTYPER software (Sequenom Inc., San Diego, USA) was applied to quantify the methylation levels for each CpG site or an average value of aggregated CpG sites.

### 2.6. Real-Time PCR Assay

Eyes were enucleated from mice during the development of EAU at different time points and tissues were carefully dissected from these eyes. TRIzol reagent (Invitrogen, Carlsbad, USA) was used to extract total RNA from the retinas and RPE-choroid complex according to the manufacturer's instructions. PrimeScript RT reagent Kit (Takara Biotechnology, Dalian, China) was used to generate complementary DNA (cDNA). Real-time PCR was performed with the real-time PCR system (ABI Prism 7500; Applied Biosystems, Foster City, USA) according to the manufacturer's instruction. For Real time-PCR analysis, each reaction was run in duplicate under the following conditions: 95°C for 10 minutes, followed by 15 seconds at 95°C for 40 cycles, and then finally incubated for 60 seconds at 60°C. Relative mRNA level was acquired by the comparative 2^−ΔΔCt^ method as previously described [34]. The sequences of primers used in this study are shown in Table 2.

### 2.7. Statistical Analysis

Data are expressed as mean ± SEM. The SPSS 22.0 software (SPSS Inc., Chicago, Illinois, USA) and GraphPad Prism 5 software (GraphPad Software Inc., San Diego, CA) were used to perform the statistical analysis. One-way ANOVA followed by Bonferroni correction was used for multiple comparisons. Pearson correlation test was used to perform a correlation analysis between CpG methylation status and the mRNA expression levels of the transcription factors at different time points during EAU development. *p* < 0.05 was considered statistically significant.

## 3. Results

### 3.1. DNA Methylation Dynamics of *Tbx21*, *Gata3*, *Rorc*, and *Foxp3* in the Retina and RPE-Choroid Complex during EAU

T-bet, GATA3, ROR*γ*t, and Foxp3 are master transcription factors for the important Th cell subsets of uveitis, including Th1, Th2, Th17, and Treg cells. Whether methylation changes of these transcription factors are involved in the pathogenesis of EAU is unclear. Therefore, we firstly investigated the dynamic DNA methylation changes of *Tbx21*, *Gata3*, *Rorc*, and *Foxp3* in the retinas and RPE-choroid complexes of EAU mice at different time points by a MassARRAY compact matrix-assisted laser desorption ionization time-of-flight (MALDI-TOF) mass spectrometer.

Following immunization of mice with the IRBP peptide, the first signs of uveitis start on the 9th day, reaching a peak on the 14th day which then gradually subside with time. The clinical and histological scores of the EAU eyes gradually returned to normal from the 21st day to the 28th day as also shown in previous studies [33, 35–37].

**Figure 1:**
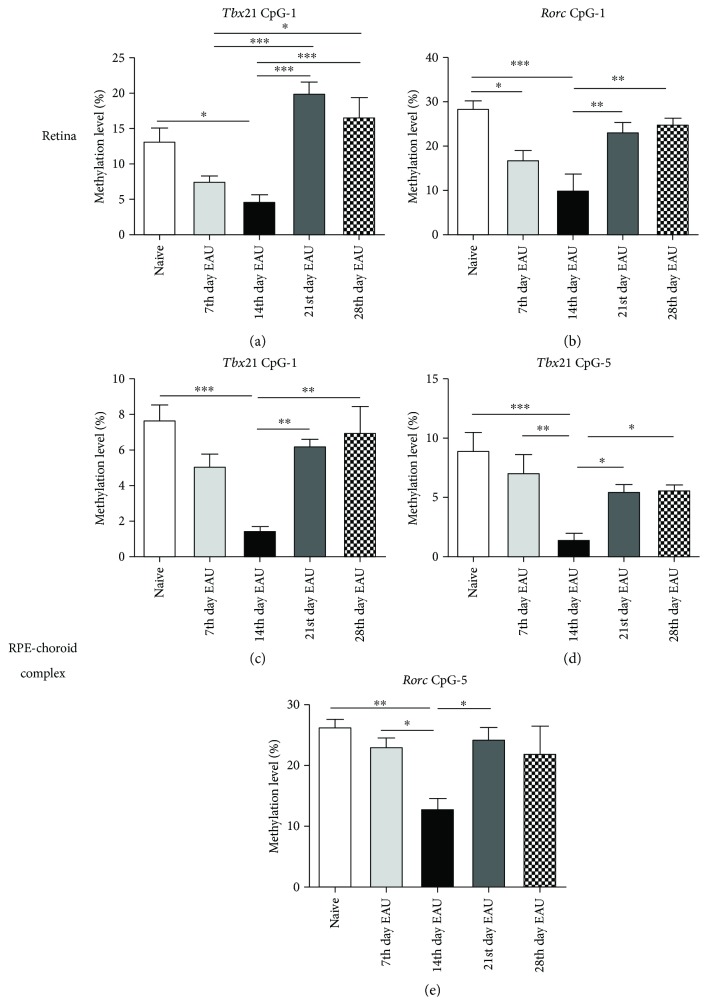
The methylation level of *Tbx21* and *Rorc* in the retinas and RPE-choroid complexes of EAU mice at different time points. The retinal methylation changes of CpG_1 for *Tbx21* (a) and *Rorc* (b) at different time points during EAU were detected with a MALDI-TOF mass spectrometer (*n* = 6, ^∗^ *p* < 0.05, ^∗∗^ *p* < 0.01, and ^∗∗∗^ *p* < 0.001). In the RPE-choroid complexes, the methylation levels of *Tbx21* in CpG_1 (c) and CpG_5 (d) and *Rorc* in CpG_5 (e) also showed a dynamic alteration with statistical difference (^∗^ *p* < 0.05, ^∗∗^ *p* < 0.01, ^∗∗∗^ *p* < 0.001, and *n* = 6). The data are shown as mean ± SEM. One-way ANOVA followed by Bonferroni correction was used to compare the methylation levels in multiple groups.

The methylation level in the retina of *Tbx21* and *Rorc* in the CpG_1 site significantly decreased and reached the nadir at the 14th day of EAU, whereafter the methylation level gradually increased again (^∗^ *p* < 0.05, ^∗∗^ *p* < 0.01, and ^∗∗∗^ *p* < 0.001) (Table 3, Figures 1(a) and 1(b)). In the RPE-choroid complexes, the methylation level of *Tbx21* in the CpG_1 and CpG_5 sites and *Rorc* in the CpG_5 site showed a similar profile (^∗^ *p* < 0.05, ^∗∗^ *p* < 0.01, and ^∗∗∗^ *p* < 0.001) (Table 4, Figures 1(c)–1(e)).

### 3.2. The mRNA Expression of *Tbx21* and *Rorc* in the Retinas and RPE-Choroid Complexes during EAU

**Figure 2:**
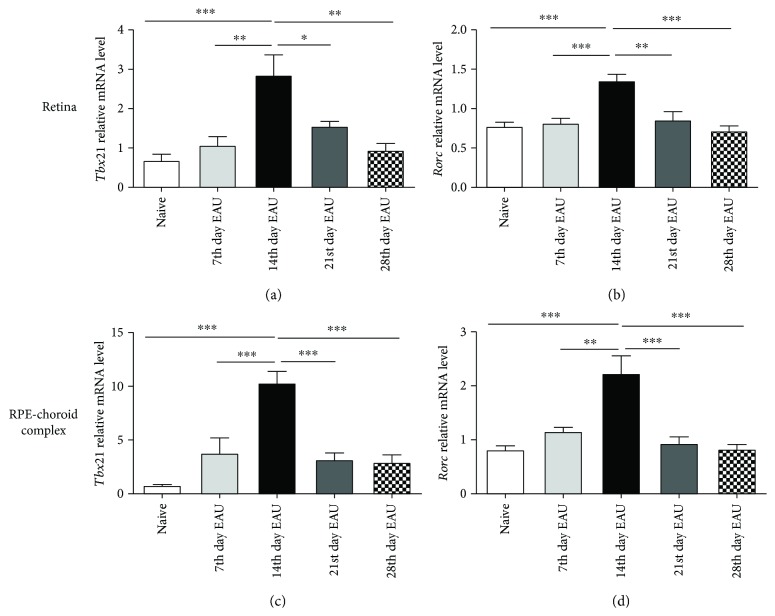
The mRNA expression of *Tbx21* and *Rorc* in the retinas and RPE-choroid complexes of EAU mice at different time points. Both the retinas and RPE-choroid complexes were dissected at different time points following IRBP peptide immunization. Then mRNA expressions of *Tbx21* and *Rorc* in the retinas (a, b) and RPE-choroid complexes (c, d) were detected with real-time PCR (^∗^ *p* < 0.05, ^∗∗^ *p* < 0.01, ^∗∗∗^ *p* < 0.001, and *n* = 6). The data are shown as mean ± SEM. One-way ANOVA followed by Bonferroni correction was used to correct for multiple comparisons.

To investigate whether the methylation change affects the gene expression, the mRNA levels of *Tbx21* and *Rorc* were detected by real-time PCR. Both in the retinas (Figures 2(a) and 2(b)) and in the RPE-choroid complexes (Figures 2(c) and 2(d)), the mRNA expression remarkably increased and reached a peak on the 14th day during the development of EAU and then the mRNA level gradually decreased in a time-dependent manner (^∗^ *p* < 0.05, ^∗∗^ *p* < 0.01, and ^∗∗∗^ *p* < 0.001). The mRNA profile resembled the changes of the DNA methylation levels shown above. These findings strongly suggest that the methylation changes were responsible for the mRNA expression alterations.

### 3.3. Correlation Analysis between CpG Methylation and mRNA Expression of *Tbx21* and *Rorc* during EAU

**Figure 3:**
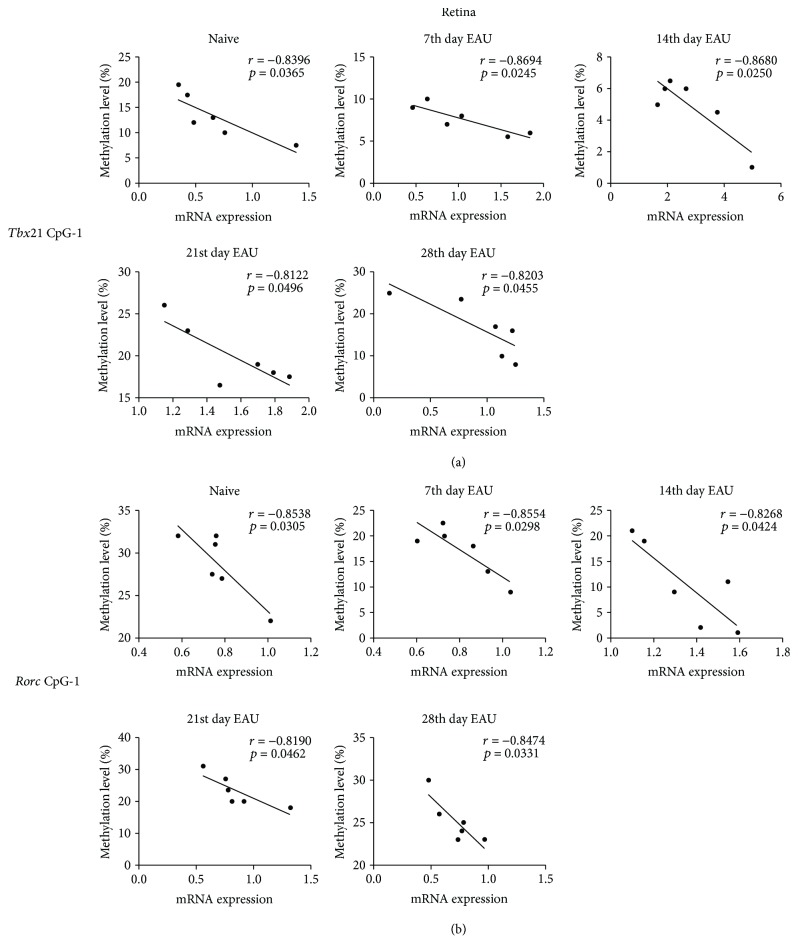
Correlation between the methylation levels of significantly changed CpG sites and the mRNA expression of *Tbx21* and *Rorc* in the retinas of EAU mice at different time points. The methylation levels of CpG-1 in *Tbx21* (a) and CpG-1 in *Rorc* (b) were negatively correlated with their mRNA expression in the retinas of the mice. Pearson correlation test was used to perform the correlation analysis between the significantly changed methylation CpG sites and the mRNA expression levels of *Tbx21* and *Rorc*.

**Figure 4:**
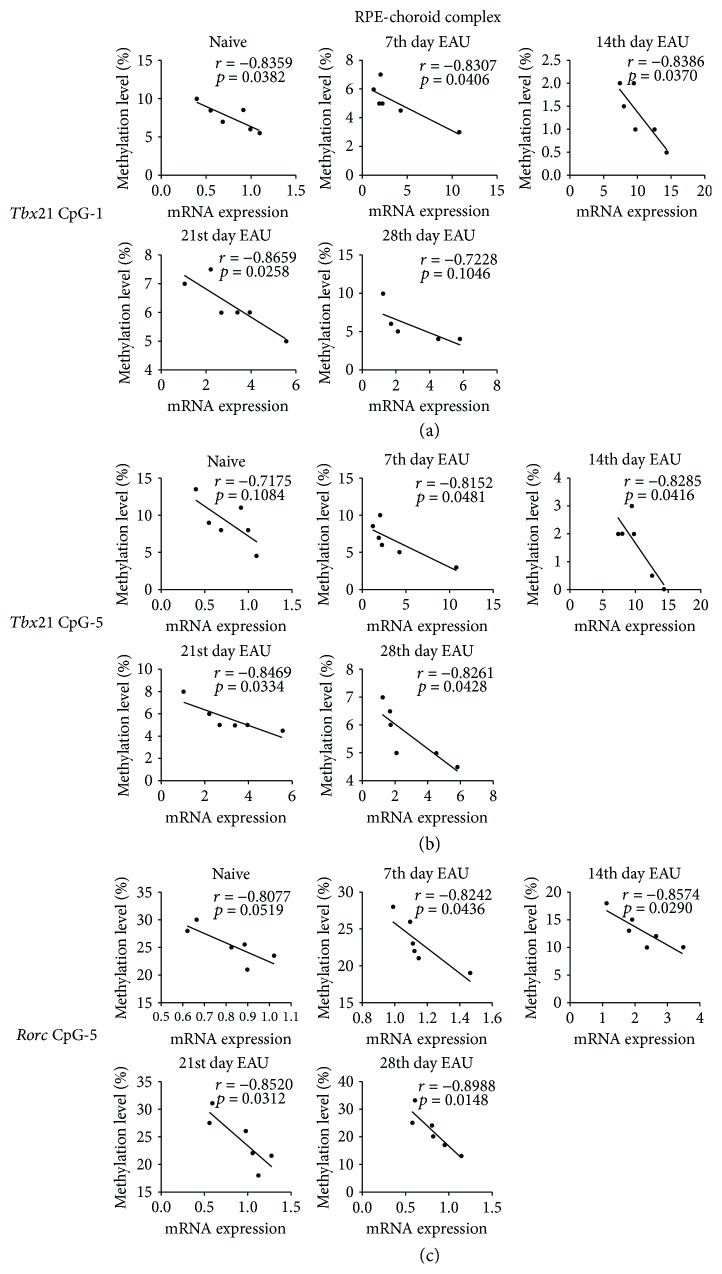
Correlation between the methylation levels of significantly changed CpG sites and the mRNA expression of *Tbx21* and *Rorc* in the RPE-choroid complexes of EAU mice at different time points. The methylation level of CpG-1 and CpG-5 in *Tbx21* (a, b) and CpG-5 in *Rorc* (c) were negatively correlated with their mRNA expression in the RPE-choroid complexes of the mice. Pearson correlation test was used to perform the correlation analysis between the significantly changed methylation CpG sites and the mRNA expression levels of *Tbx21*and *Rorc*.

To investigate the association between CpG methylation and the mRNA expression of these genes during EAU development, correlation analysis was performed to analyze the relationship between the CpG-1 site and the mRNA expression of *Tbx21* and *Rorc* in the retinas of EAU mice at different time points during EAU development. We found that the methylation level of the CpG-1 site showed a negative correlation with the mRNA expression of both *Tbx21* (Figure 3(a)) and *Rorc* (Figure 3(b)) in the retinas of the mice at different time points during EAU development. In addition, the methylation levels of CpG-1, CpG-5 of *Tbx21* (Figures 4(a) and 4(b)), and CpG-5 of *Rorc* (Figure 4(c)) were also negatively correlated with their mRNA expression in the RPE-choroid complex of the mice during EAU.

### 3.4. The mRNA Expression of the Signature Cytokines of Th1 and Th17 Cells in the Retinas and RPE-Choroid Complexes during EAU

**Figure 5:**
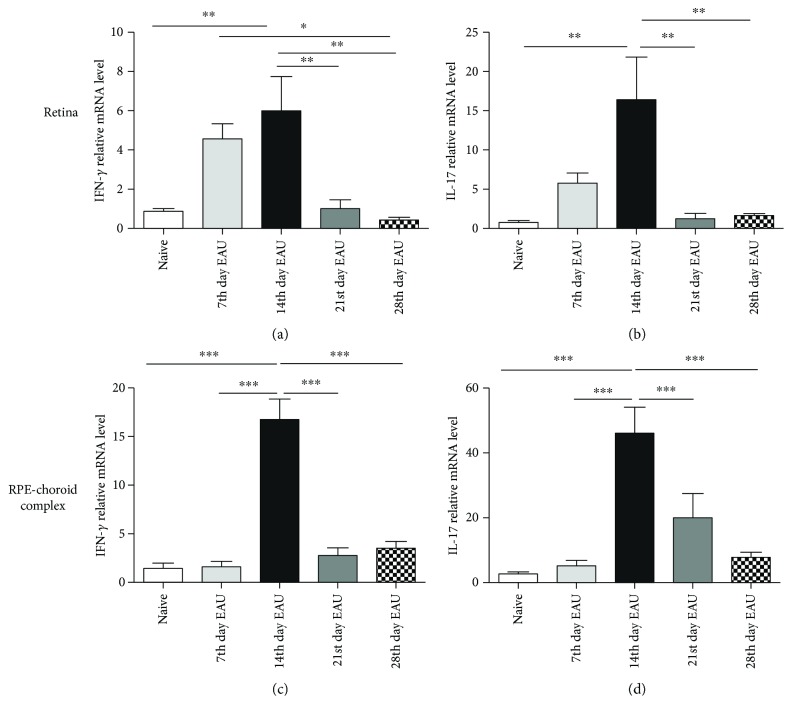
The mRNA expression of IFN-*γ* and IL-17 in the retinas and RPE-choroid complexes of EAU mice at different time points. The mRNA expressions of IFN-*γ* and IL-17 in the retinas (a, b) and RPE-choroid complexes (c, d) were detected with real-time PCR at different time points. (^∗^ *p* < 0.05, ^∗∗^ *p* < 0.01, ^∗∗∗^ *p* < 0.001, and *n* = 6). The data are shown as mean ± SEM. One-way ANOVA followed by Bonferroni correction was applied to compare the mRNA expressions in multiple groups.

Previous studies indicated that aberrant epigenetic modifications may be closely associated with abnormal production of inflammatory cytokines in several autoimmune diseases [38]. Hence, we investigated whether the DNA methylation changes affected the production of the signature cytokines of Th1 and Th17 cells in our EAU model. The mRNA expressions of IFN-*γ* and IL-17 were measured by real-time PCR. Our data showed that the mRNA expression of IFN-*γ* and IL-17 gradually increased during the development of EAU and reached its peak on the 14th day and then decreased in both the retinas (Figures 5(a) and 5(b)) and the RPE-choroid complexes (Figure 5(c) and 5(d)) (^∗^ *p* < 0.05, ^∗∗^ *p* < 0.01, and ^∗∗∗^ *p* < 0.001). These findings suggest that the decreased DNA methylation observed during EAU is associated with an increased production of proinflammatory cytokines.

### 3.5. The mRNA Expression of DNMT1, DNMT3a, and DNMT3b during EAU

**Figure 6:**
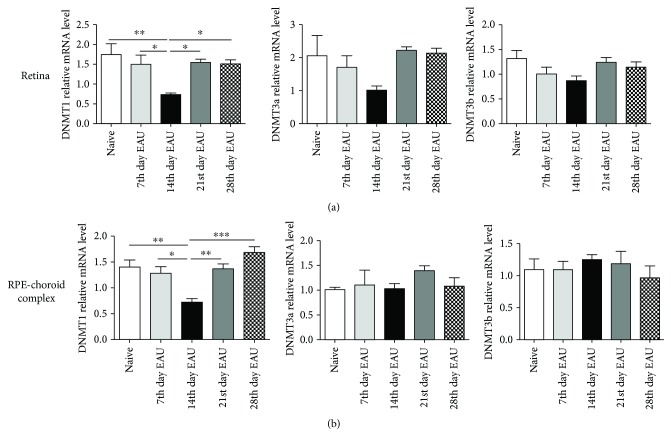
The mRNA expression of DNMT1, DNMT3a, and DNMT3b in the retina and RPE-choroid complex of EAU mice at different time points. Total RNA was extracted from the retinas and RPE-choroid complexes of EAU mice at different time points. The mRNA expression of DNMT1, DNMT3a, and DNMT3b in the retina (a) and RPE-choroid complex (b) was detected with real-time PCR (^∗^ *p* < 0.05, ^∗∗^ *p* < 0.01, ^∗∗∗^ *p* < 0.001, and *n* = 6). The data are shown as mean ± SEM. One-way ANOVA followed by Bonferroni correction was used to analyze the mRNA expressions.

DNA methyltransferases (DNMTs) are specific catalytically active enzymes which modulate the accessibility of DNA to transcriptional elements, thus to enhance DNA methylation. To study the involvement of DNMTs in the regulation of methylation of *Tbx21* and *Rorc*, we examined the expression changes of DNMT1, DNMT3a, and DNMT3b in both the retinas and RPE-choroid complexes of EAU mice at different time points. The results showed that in the retinas, DNMT1 expression revealed a dynamic change corresponding to the methylation of *Tbx21* and *Rorc*, which was not observed for DNMT3a or DNMT3b (Figure 6(a)) (^∗^ *p* < 0.05, ^∗^ *p* < 0.01). DNMT1 expression with time showed similar results in the RPE-choroid complex as seen in the retina (Figure 6(b)) (^∗^ *p* < 0.05, ^∗^ *p* < 0.01, and ^∗∗∗^ *p* < 0.001).

## 4. Discussion

This study shows significant DNA methylation dynamics corresponding with mRNA expression changes of *Tbx21* and *Rorc* in the inflamed tissue of mice undergoing experimental autoimmune uveitis. Decreased DNA methylation observed during EAU was associated with an increased expression of the Th1/Th17 signature cytokines IFN-*γ* and IL-17. We furthermore provide preliminary evidence for a role of DNMT1 in the regulation of methylation changes of *Tbx21* and *Rorc* during EAU. Taken together, the data suggest that aberrant epigenetic changes of these Th1 and Th17 transcription factors may be involved in the pathogenesis of EAU.

Accumulating evidence indicates that epigenetic modifications may play an important role in the pathogenesis of autoimmune disease [26–30]. Dysfunction of immune responses and overproduction of inflammatory cytokines in autoimmune disease has been shown to be associated with aberrant epigenetic modifications [38]. Our study, where we showed that DNA methylation dynamics of *Tbx21* and *Rorc* correspond to their gene expression and whereby methylation of these factors is related to the expression of IFN-*γ* and IL-17, supports these findings. Our findings are in disagreement with a previous study that failed to find aberrant methylation of *Tbx21* or *Rorc* in CD4^+^ T cells from patients with VKH uveitis [30]. In the latter study, hypermethylation of *Gata3* was noted in VKH patients, while in our EAU model, we did not show any changes in the *Gata3* methylation levels with time. Abnormal methylation changes of *Foxp3* have been reported with several autoimmune diseases [28, 39], which could also not be confirmed in our EAU model. The discrepancies may be due to differences between the roles of these various transcription factors in the pathogenesis of mouse autoimmune models versus human autoimmune disease. A possible mechanism of abnormal methylation changes of *Tbx21* and *Rorc* in EAU may be reflected by the activation of Th1 and Th17 cells. It has been well studied that Th1 and Th17 cells play crucial roles in the pathological process of EAU. In the present study, we noted dynamic methylation changes of *Tbx21* and *Rorc*, the signature transcription factors of Th1 cells and Th17 cells, supporting our finding that the alteration of methylation status of *Tbx21* and *Rorc* is closely associated with the disease progression of uveitis.

The methylation level of specific autoimmunity-related genes as seen in various autoimmune disorders, including SLE and RA, may be associated with changes in the expression of enzymes regulating DNA methylation such as the DNMTs [22, 40]. The mRNA expressions of DNMT1 and DNMT3a were significantly decreased in the peripheral blood mononuclear cells (PBMCs) of SLE patients as compared to healthy subjects [41]. An aberrant mRNA expression of DNMT1 was also observed in PBMCs from vitiligo patients [42]. These observations are confirmed by our findings showing dynamic changes in DNMT1 mRNA expression during EAU. To further confirm the regulation of DNA methylation changes by DNMT1, chromatin immunoprecipitation (ChIP) analysis of DNMT1 enrichment in the promoter of *Tbx21* and *Rorc* genes needs to be performed in future studies.

It is well known that the molecular and cellular events are not static during the different clinical stages of an autoimmune disease. During disease development, dynamic DNA methylation changes regulate gene expression and in turn determine the molecular and cellular events. Hence, the degree of the methylation of certain genes may serve as sensitive biomarkers for both early diagnosis and assessment of disease status. Moreover, assessment of DNA methylation dynamics can also lead to the development of novel intervention targets for autoimmune disease.

We realize that our study has a number of limitations that should be addressed in future research. Firstly, a technical limitation of the MassARRAY system cannot be ignored. DNA methylation often occurs in the CpG islands in the promoter region which subsequently affects the transcription of associated genes. Therefore, we designed the methylation detection primers according to the DNA sequence of the promoter region which contains the CpG islands of each gene. However, the coverage of methylation detection primers was limited to less than 500 bp, resulting in a limited region of the promoter that can be covered with this method [30, 43, 44]. Moreover, methylation analysis of a limited number of CpG sites may not be sufficient to study the overall epigenetic regulatory effects on such a gene. Therefore, further studies with other detection techniques, such as bisulfite sequencing or epigenome-wide association studies (EWAS), are necessary to address the exact role of epigenetic regulatory effects in the pathogenesis of experimental as well as clinical uveitis. Our findings were performed in mice and one should be careful to extrapolate these findings to humans. Additionally, although we suggest that a local increase of DNMT1 may be used to block cytokine expression during autoimmune disease, further extensive research is needed to investigate whether this is feasible and whether treatment can be restricted to specific genes.

## 5. Conclusions

In summary, we show for the first time that dynamic methylation changes of the important T cell transcription regulation factors T-bet and ROR*γ*t can be detected during the process of EAU. Decreased DNA methylation changes were associated with a higher mRNA expression. We furthermore provide evidence showing that local DNA methylation is correlated with DNMT1 mRNA expression which opens new venues for the treatment of autoimmune diseases such as uveitis.

## Figures and Tables

**Table 1:** Sequences of the primers for methylation detection (5′ to 3′).

Gene	Primer	Sequence	Product length	Location
*Tbx21*	Forward	aggaagagagTTTGAAGTTGTAGGTTGTTGAGTTT	193 bp	−1806~−1998
	Reverse	cagtaatacgactcactatagggagaaggctAAATCATCCTTTCCTCTACACCAAAA		
*Gata3*	Forward	aggaagagagGGTTGTAGTTTTTTTGTGTTGAGGA	323 bp	−895~−1217
	Reverse	cagtaatacgactcactatagggagaaggctATCACAAATCACAATACCAACCTAAAT		
*Rorc*	Forward	aggaagagagTTGGTTTTTAGGTGTGAGTAGGTTT	281 bp	−1459~−1739
	Reverse	cagtaatacgactcactatagggagaaggctAAAACTATTCCCCAATTCCTTTCTA		
*Foxp3*	Forward	aggaagagagGGTTTTGATTATATGTTTGTTTGTTTTT	353 bp	−1496~−1848
	Reverse	cagtaatacgactcactatagggagaaggctTCCTAAACTACCTACTCTCCTTACACA		

**Table 2:** Sequences of the primers for real-time PCR (5′ to 3′).

Primer	Accession number	Forward	Reverse
*Tbx21*	NM_019507.2	TCAACCAGCACCAGACAGAG	CCACATCCACAAACATCCTG
*Rorc*	NM_011281.3	GAAGGCAAATACGGTGGTGT	AGAGGGCAATCTCATCCTCA
*IFN-γ*	NM_008337.4	TCAAGTGGCATAGATGTGGAAGAA	TGGCTCTGCAGGATTTTCATG
*IL-17*	NM_010552.3	CTCAACCGTTCCACGTCACCCT	CCAGCTTTCCCTCCGCATT
*DNMT1*	XM_011242393.2	TGGTGTTGTCTACCGACTGG	CAGGGTCTCGTTCACAGGAT
*DNMT3a*	XM_006514956.3	GTACCGCAAAGCCATCTACG	TTGTTCTGCACTTCCACAGC
*DNMT3b*	XM_006498689.3	ACTTGGTGATTGGTGGAAGC	AGAAGAATGGACGGTTGTCG
*GAPDH*	NM_001289726.1	GTATGACTCCACTCACGGCAAA	GGTCTCGCTCCTGGAAGATG

**Table 3:** Methylation levels of Tbx21, Gata3, Rorc, and Foxp3 in the retinas of EAU mice at different time points.

Gene	CpG site	Naïve methylation level (%)	EAU 7th-day methylation level (%)	EAU 14th-day methylation level (%)	EAU 21st-day methylation level (%)	EAU 28th-day methylation level (%)
*Tbx21*	CpG_1	9.92 ± 2.27^#^	7.58 ± 1.74^#^	4.67 ± 2.40^#^	20.00 ± 3.70^#^	14.92 ± 8.14^#^
	CpG_3	8.07 ± 2.09	9.66 ± 4.07	6.17 ± 2.80	5.58 ± 6.42	5.00 ± 7.48
	CpG_5	6.58 ± 4.59	3.58 ± 3.00	3.67 ± 2.86	4.25 ± 0.41	5.67 ± 4.08
	CpG_6, 7	4.25 ± 1.41	2.50 ± 2.35	2.42 ± 1.43	4.53 ± 1.02	4.10 ± 2.48
	CpG_8	7.58 ± 1.07	7.67 ± 1.17	6.92 ± 1.88	8.75 ± 0.99	9.25 ± 1.55

*Gata3*	CpG_1	8.00 ± 2.28	7.00 ± 6.56	8.33 ± 3.43	5.50 ± 1.87	10.33 ± 2.94
	CpG_2	17.00 ± 7.40	33.17 ± 21.32	26.42 ± 13.28	16.17 ± 8.45	19.50 ± 4.85
	CpG_6, 7, 8	13.00 ± 3.11	15.33 ± 6.89	14.83 ± 8.04	12.75 ± 5.52	17.00 ± 5.68
	CpG_9	2.58 ± 1.24	1.50 ± 1.97	2.00 ± 1.45	1.50 ± 1.22	1.00 ± 0.71
	CpG_10, 11	14.58 ± 1.69	9.60 ± 3.58	22.92 ± 10.87	26.80 ± 24.51	30.58 ± 15.79
	CpG_14	2.92 ± 3.01	4.40 ± 5.18	3.75 ± 3.17	2.42 ± 1.36	1.17 ± 1.16
	CpG_16	5.57 ± 1.97	2.40 ± 3.31	4.75 ± 2.54	5.50 ± 0.77	4.75 ± 2.82

*Rorc*	CpG_1	28.58 ± 3.90^#^	16.92 ± 4.98^#^	10.00 ± 8.99^#^	23.25 ± 4.96^#^	25.00 ± 2.92^#^
	CpG_2	72.60 ± 4.77	64.75 ± 1.29	74.25 ± 3.72	71.67 ± 5.32	56.25 ± 22.09
	CpG_3, 4	11.25 ± 4.83	24.50 ± 30.21	15.42 ± 3.64	21.46 ± 20.89	56.50 ± 3.32
	CpG_5	38.75 ± 5.96	36.41 ± 9.09	33.08 ± 8.37	46.30 ± 11.80	51.00 ± 11.34
	CpG_6	36.75 ± 4.77	31.92 ± 2.15	31.25 ± 9.79	31.08 ± 4.43	33.50 ± 3.41

*Foxp3*	CpG_2	81.58 ± 5.15	78.83 ± 9.30	79.00 ± 3.78	80.00 ± 5.48	85.38 ± 5.50
	CpG_3	89.33 ± 19.74	97.08 ± 5.12	98.75 ± 1.99	99.80 ± 0.45	90.50 ± 19.00
	CpG_5	62.50 ± 33.89	64.25 ± 23.35	73.75 ± 21.14	71.00 ± 18.57	75.33 ± 4.51

# indicates the data of the group showing a statistically significant (*p* < 0.05, *p* < 0.01, and *p* < 0.001) difference compared with other groups among the five groups (one-way ANOVA followed by Bonferroni correction was used to compare the methylation levels in multiple groups).

**Table 4:** Methylation levels of Tbx21, Gata3, Rorc, and Foxp3 in the RPE-choroid complex of EAU mice at different time points.

Gene	CpG site	Naïve methylation level (%)	EAU 7th-day methylation level (%)	EAU 14th-day methylation level (%)	EAU 21st-day methylation level (%)	EAU 28th-day methylation level (%)
*Tbx21*	CpG_1	7.70 ± 1.89^#^	5.13 ± 1.31	1.50 ± 0.50^#^	6.25 ± 0.88^#^	7.00 ± 3.52^#^
	CpG_3	13.58 ± 4.65	9.70 ± 5.83	5.42 ± 5.92	11.58 ± 6.87	6.58 ± 5.55
	CpG_5	9.00 ± 3.41^#^	7.13 ± 3.01^#^	1.50 ± 1.22^#^	5.58 ± 1.28^#^	5.67 ± 0.98^#^
	CpG_6, 7	4.75 ± 1.33	4.40 ± 1.98	2.58 ± 0.97	4.00 ± 1.26	2.58 ± 0.80
	CpG_8	11.25 ± 2.50	8.25 ± 3.84	13.25 ± 2.93	12.50 ± 1.05	12.92 ± 2.13

*Gata3*	CpG_1	8.42 ± 3.20	1.50 ± 2.12	4.33 ± 2.25	4.40 ± 6.10	10.83 ± 5.23
	CpG_2	25.30 ± 19.23	39.00 ± 13.30	32.17 ± 9.52	22.25 ± 5.76	21.25 ± 5.35
	CpG_6, 7, 8	11.00 ± 2.28	23.67 ± 7.53	13.17 ± 7.78	21.25 ± 10.97	22.25 ± 12.32
	CpG_9	1.67 ± 2.23	1.90 ± 3.05	2.83 ± 1.51	1.17 ± 0.52	0.83 ± 0.52
	CpG_10, 11	12.50 ± 1.26	10.64 ± 5.23	32.17 ± 19.31	32.92 ± 20.45	33.25 ± 15.89
	CpG_14	8.00 ± 4.72	8.08 ± 9.35	10.08 ± 4.43	2.92 ± 1.74	1.83 ± 1.51
	CpG_16	7.50 ± 3.77	3.58 ± 5.61	5.83 ± 1.69	4.58 ± 1.43	2.00 ± 1.70

*Rorc*	CpG_1	13.13 ± 2.39	5.50 ± 6.60	4.40 ± 3.23	7.42 ± 3.01	6.20 ± 3.42
	CpG_2	33.58 ± 10.93	37.83 ± 14.77	39.17 ± 7.13	16.28 ± 13.01	18.61 ± 14.38
	CpG_3, 4	13.33 ± 4.12	10.83 ± 6.38	9.30 ± 5.38	14.00 ± 5.66	10.50 ± 0.71
	CpG_5	26.40 ± 2.58^#^	23.17 ± 3.31^#^	13.00 ± 3.46^#^	24.33 ± 4.71^#^	22.00 ± 8.87
	CpG_6	18.33 ± 7.74	20.17 ± 7.33	10.75 ± 4.67	11.42 ± 4.04	13.10 ± 3.50

*Foxp3*	CpG_2	74.33 ± 6.57	70.00 ± 9.25	79.83 ± 4.97	79.00 ± 7.55	86.50 ± 1.41
	CpG_3	94.42 ± 6.79	99.83 ± 0.41	99.33 ± 1.63	91.25 ± 17.50	88.50 ± 16.26
	CpG_5	78.90 ± 4.38	56.17 ± 9.09	72.58 ± 12.82	72.00 ± 11.31	59.00 ± 5.65

# indicates the data of the group showing a statistically significant (*p* < 0.05, *p* < 0.01, and *p* < 0.001) difference compared with other groups among the five groups (one-way ANOVA followed by Bonferroni correction was used to compare the methylation levels in multiple groups).

## Data Availability

The data used to support the findings of this study are included within the article.

## Supplementary Materials

Supplementary Materials Table S1: location information of methylation detection primers and CpG islands for Tbx21, Gata3, Rorc, and Foxp3. Figure S1: the location information of methylation detection primers of Tbx21, Gata3, Rorc, and Foxp3. The methylation primer-detected gene region of Tbx21 was located between 1806 and 1998, the detected gene region of Gata3 was located between 895 and 1217, the detected gene region of Rorc was located between 1459 and 1739, and the detected gene region of Foxp3 was located between 1496 and 1848 from the transcription start site (TSS).

## References

[1] Suttorp-Schulten M. S., Rothova A. (1996). The possible impact of uveitis in blindness: a literature survey. *The British Journal of Ophthalmology*.

[2] Nussenblatt R. B. (1990). The natural history of uveitis. *International Ophthalmology*.

[3] Nussenblatt R. B. (1991). Proctor lecture. Experimental autoimmune uveitis: mechanisms of disease and clinical therapeutic indications. *Investigative Ophthalmology & Visual Science*.

[4] Caspi R. R., Roberge F. G., Chan C. C. (1988). A new model of autoimmune disease. Experimental autoimmune uveoretinitis induced in mice with two different retinal antigens. *Journal of Immunology*.

[5] Jiang H. R., Lumsden L., Forrester J. V. (1999). Macrophages and dendritic cells in IRBP-induced experimental autoimmune uveoretinitis in B10RIII mice. *Investigative Ophthalmology & Visual Science*.

[6] Kim J., Park J. A., Lee E. Y., Lee Y. J., Song Y. W., Lee E. B. (2010). Imbalance of Th17 to Th1 cells in Behçet’s disease. *Clinical and Experimental Rheumatology*.

[7] Chi W., Yang P., Li B. (2007). IL-23 promotes CD4+ T cells to produce IL-17 in Vogt-Koyanagi-Harada disease. *The Journal of Allergy and Clinical Immunology*.

[8] Chen L., Yang P., Zhou H. (2008). Diminished frequency and function of CD4^+^CD25^high^ regulatory T cells associated with active uveitis in Vogt-Koyanagi-Harada syndrome. *Investigative Ophthalmology & Visual Science*.

[9] Raziuddin S., Al-Dalaan A., Bahabri S., Siraj A. K., al-Sedairy S. (1998). Divergent cytokine production profile in Behçet’s disease. Altered Th1/Th2 cell cytokine pattern. *The Journal of Rheumatology*.

[10] Miller S. A., Weinmann A. S. (2009). Common themes emerge in the transcriptional control of T helper and developmental cell fate decisions regulated by the T-box, GATA and ROR families. *Immunology*.

[11] Oda J. M. M., Hirata B. K. B., Guembarovski R. L., Watanabe M. (2013). Genetic polymorphism in *FOXP3* gene: imbalance in regulatory T-cell role and development of human diseases. *Journal of Genetics*.

[12] Liao D., Hou S., Zhang J. (2015). Copy number variants and genetic polymorphisms in *TBX21*, *GATA3*, *Rorc*, *Foxp3* and susceptibility to Behcet’s disease and Vogt-Koyanagi-Harada syndrome. *Scientific Reports*.

[13] Bai L., Liu Y., Hou S., Liao D., Kijlstra A., Yang P. (2016). Association of T-bet, GATA-3, RORC, and FOXP3 copy number variations with acute anterior uveitis with or without ankylosing spondylitis in Chinese Han. *Investigative Ophthalmology & Visual Science*.

[14] Li B., Yang P., Chu L. (2007). T-bet expression in the iris and spleen parallels disease expression during endotoxin-induced uveitis. *Graefe's archive for clinical and experimental ophthalmology*.

[15] Takase H., Yu C. R., Ham D. I. (2006). Inflammatory processes triggered by TCR engagement or by local cytokine expression: differences in profiles of gene expression and infiltrating cell populations. *Journal of Leukocyte Biology*.

[16] Kerr E. C., Raveney B. J. E., Copland D. A., Dick A. D., Nicholson L. B. (2008). Analysis of retinal cellular infiltrate in experimental autoimmune uveoretinitis reveals multiple regulatory cell populations. *Journal of Autoimmunity*.

[17] Dawson M. A., Kouzarides T. (2012). Cancer epigenetics: from mechanism to therapy. *Cell*.

[18] Jones P. A. (2012). Functions of DNA methylation: islands, start sites, gene bodies and beyond. *Nature Reviews Genetics*.

[19] Wilson C. B., Makar K. W., Shnyreva M., Fitzpatrick D. R. (2005). DNA methylation and the expanding epigenetics of T cell lineage commitment. *Seminars in Immunology*.

[20] Zhao S., Wang Y., Liang Y. (2011). MicroRNA-126 regulates DNA methylation in CD4+ T cells and contributes to systemic lupus erythematosus by targeting DNA methyltransferase 1. *Arthritis and Rheumatism*.

[21] Deng Y., Tsao B. P. (2014). Advances in lupus genetics and epigenetics. *Current Opinion in Rheumatology*.

[22] Liu C. C., Fang T. J., Ou T. T. (2011). Global DNA methylation, DNMT1, and MBD2 in patients with rheumatoid arthritis. *Immunology Letters*.

[23] Calabrese R., Zampieri M., Mechelli R. (2012). Methylation-dependent *PAD2* upregulation in multiple sclerosis peripheral blood. *Multiple Sclerosis Journal*.

[24] Belot M. P., Fradin D., Mai N. (2013). CpG methylation changes within the IL2RA promoter in type 1 diabetes of childhood onset. *PLoS One*.

[25] Gu T., Falhammar H., Gu H. F., Brismar K. (2014). Epigenetic analyses of the insulin-like growth factor binding protein 1 gene in type 1 diabetes and diabetic nephropathy. *Clinical Epigenetics*.

[26] Qiu Y., Zhu Y., Yu H. (2017). Ocular Behcet's disease is associated with aberrant methylation of interferon regulatory factor 8 (IRF8) in monocyte-derived dendritic cells. *Oncotarget*.

[27] Qiu Y., Yu H., Zhu Y. (2017). Hypermethylation of interferon regulatory factor 8 (IRF8) confers risk to Vogt-Koyanagi-Harada disease. *Scientific Reports*.

[28] Kennedy A., Schmidt E. M., Cribbs A. P. (2014). A novel upstream enhancer of *FOXP3*, sensitive to methylation-induced silencing, exhibits dysregulated methylation in rheumatoid arthritis Treg cells. *European Journal of Immunology*.

[29] Wang Z., Zheng Y., Hou C. (2013). DNA methylation impairs TLR9 induced Foxp3 expression by attenuating IRF-7 binding activity in fulminant type 1 diabetes. *Journal of Autoimmunity*.

[30] Zhu Y., Yu H., Qiu Y. (2017). Promoter hypermethylation of GATA3, IL-4, and TGF-*β* confers susceptibility to Vogt-Koyanagi-Harada disease in Han Chinese. *Investigative Ophthalmology & Visual Science*.

[31] Xie W., Schultz M. D., Lister R. (2013). Epigenomic analysis of multilineage differentiation of human embryonic stem cells. *Cell*.

[32] Meissner A., Mikkelsen T. S., Gu H. (2008). Genome-scale DNA methylation maps of pluripotent and differentiated cells. *Nature*.

[33] Qiu Y., Tao L., Zheng S. (2016). AAV8-mediated angiotensin-converting enzyme 2 gene delivery prevents experimental autoimmune uveitis by regulating MAPK, NF-*κ*B and STAT3 pathways. *Scientific Reports*.

[34] Qiu Y., Shil P. K., Zhu P. (2014). Angiotensin-converting enzyme 2 (ACE2) activator diminazene aceturate ameliorates endotoxin-induced uveitis in mice. *Investigative Ophthalmology & Visual Science*.

[35] Yang H., Zheng S., Qiu Y. (2014). Activation of liver X receptor alleviates ocular inflammation in experimental autoimmune uveitis. *Investigative Ophthalmology & Visual Science*.

[36] Sun M., Yang P., Du L., Zhou H., Ren X., Kijlstra A. (2010). Contribution of CD4+CD25+ T cells to the regression phase of experimental autoimmune uveoretinitis. *Investigative Ophthalmology & Visual Science*.

[37] Sun M., Yang P., Du L. (2010). Increased regulatory T cells in spleen during experimental autoimmune uveoretinitis. *Ocular Immunology and Inflammation*.

[38] Zhao M., Wang Z., Yung S., Lu Q. (2015). Epigenetic dynamics in immunity and autoimmunity. *The International Journal of Biochemistry & Cell Biology*.

[39] Wang Y. Y., Wang Q., Sun X. H. (2014). DNA hypermethylation of the forkhead box protein 3 (FOXP3) promoter in CD4+ T cells of patients with systemic sclerosis. *The British Journal of Dermatology*.

[40] Balada E., Ordi-Ros J., Vilardell-Tarres M. (2007). DNA methylation and systemic lupus erythematosus. *Annals of the New York Academy of Sciences*.

[41] Nawrocki M. J., Majewski D., Puszczewicz M., Jagodzinski P. P. (2017). Decreased mRNA expression levels of DNA methyltransferases type 1 and 3A in systemic lupus erythematosus. *Rheumatology International*.

[42] Zhao M., Gao F., Wu X., Tang J., Lu Q. (2010). Abnormal DNA methylation in peripheral blood mononuclear cells from patients with vitiligo. *The British Journal of Dermatology*.

[43] Fuchikami M., Morinobu S., Segawa M. (2011). DNA methylation profiles of the brain-derived neurotrophic factor (BDNF) gene as a potent diagnostic biomarker in major depression. *PLoS One*.

[44] Shinojima Y., Terui T., Hara H. (2010). Identification and analysis of an early diagnostic marker for malignant melanoma: ZAR1 intra-genic differential methylation. *Journal of Dermatological Science*.

