# Supplementary material for: Dynamic DNA Methylation Changes of Tbx21 and Rorc during Experimental Autoimmune Uveitis in Mice
Source: Mediators Inflamm. 2018 Sep 4;2018:9129163. doi: 10.1155/2018/9129163 (PMC6142759; doi:10.1155/2018/9129163)

Table S1. Location information of methylation detection primers and CpG islands for *Tbx21*, *Gata3*, *Rorc* and *Foxp3*

Gene	CpG island location (from TSS)	Detect region of methylation primers (from TSS)
Tbx21	From 899 to 2020	From 1806 to 1998
Gata3	From 957 to 1181	From 895 to 1217
Rorc	From 1516 to 1574	From 1459 to 1739
Foxp3	From 1560 to 1639	From 1496 to 1848

Tbx21

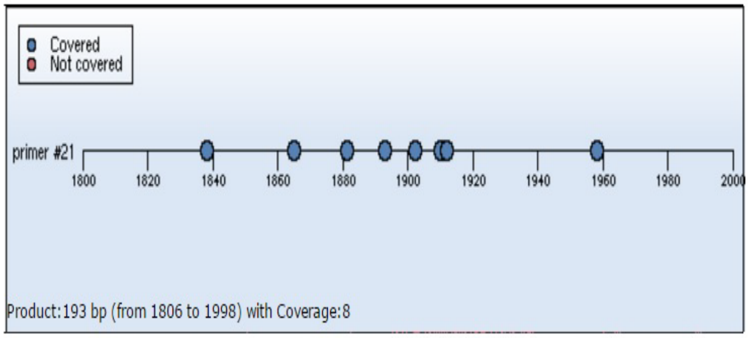

Gata3

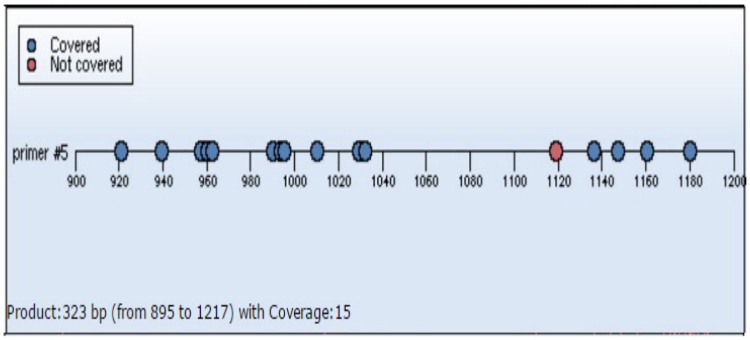

Rorc

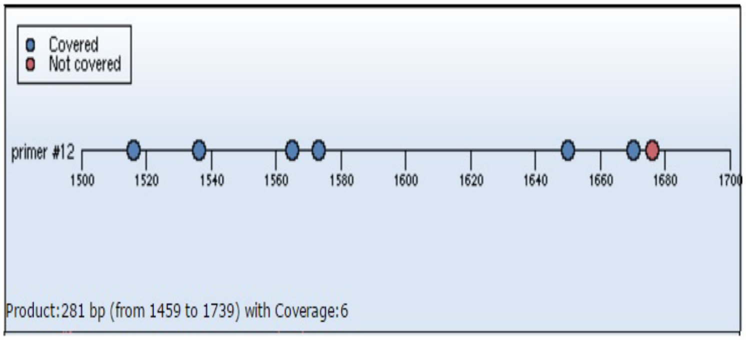

Foxp3

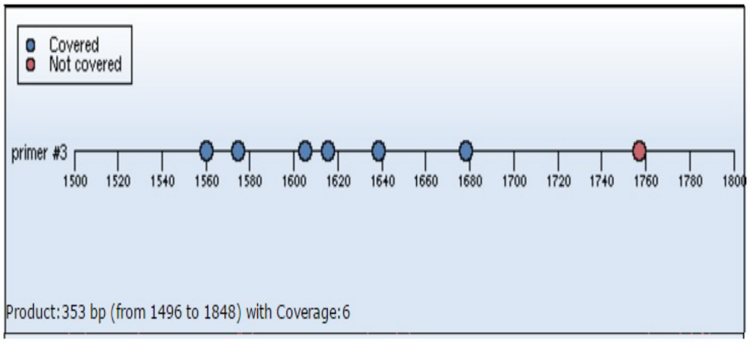

Supplement: Supplementary Materials — Table S1: location information of methylation detection primers and CpG islands for Tbx21, Gata3, Rorc, and Foxp3. Figure S1: the location information of methylation detection primers of Tbx21, Gata3, Rorc, and Foxp3. The methylation primer-detected gene region of Tbx21 was located between 1806 and 1998, the detected gene region of Gata3 was located between 895 and 1217, the detected gene region of Rorc was located between 1459 and 1739, and the detected gene region of Foxp3 was located between 1496 and 1848 from the transcription start site (TSS). [file 9129163.f1.pdf]
